# Music-based interventions in the treatment of eating disorders: a scoping review

**DOI:** 10.3389/fpsyt.2025.1660696

**Published:** 2025-09-24

**Authors:** Annie Heiderscheit, Julia Gawronska, Jodie Bloska, Anya Ragnhildstveit, Thandi Milton, Sharon A. S. Neufeld

**Affiliations:** ^1^ Cambridge Institute for Music Therapy Research, Anglia Ruskin University, Cambridge, United Kingdom; ^2^ Centre for Health, Performance, and Wellbeing, Anglia Ruskin University, Cambridge, United Kingdom; ^3^ Department of Psychiatry, University of Cambridge, Cambridge, United Kingdom; ^4^ Department of Paediatrics, Cambridge University Hospitals National Health Service (NHS) Foundation Trust, Cambridge, United Kingdom

**Keywords:** music therapy, music-based interventions, eating disorders, scoping review, eating disorder treatment

## Abstract

**Introduction:**

Eating disorders (EDs) are potentially life-threatening conditions characterized by persistent eating- and body-related disturbances that negatively impact nutritional intake, psychological well-being, and physical health. While psychological therapies remain the primary treatment for patients with EDs, music-based interventions (MBIs) show significant promise for addressing symptoms in clinical practice. However, little is known about the methods employed in these interventions or their effectiveness in addressing or improving ED outcomes.

**Methods:**

Following PRISMA-ScR guidelines, we conducted a scoping review of the literature. Eight electronic databases were systematically queried from inception to May 2025 for studies evaluating MBIs in patients engaged in ED treatment. Data regarding study design, sample characteristics, intervention components, and primary outcomes were extracted and analysed utilising either reflexive thematic analysis or descriptive statistics. The quality of intervention reporting was additionally examined utilising the established MBI reporting guidelines.

**Results:**

Of the 114 articles screened, 21 met inclusion criteria. The final sample comprised 10 case studies, 6 qualitative studies, 4 quantitative studies, and 1 mixed methods study, with all but one conducted in high-income countries. Settings included inpatient (*n* = 12), outpatient (*n* = 7), and combined (*n* = 2) ED programs. Most MBIs involved music therapy (*n* = 18), while others incorporated vibroacoustic therapy (*n* = 1), background music during mealtimes (*n* = 1), and group singing (*n* = 1). Delivery formats encompassed individual (*n* = 14), group (*n* = 5), and hybrid (*n* = 1) sessions, or were not indicated. Qualitative synthesis identified seven themes reflecting symptom management, self-development, and treatment engagement. Quantitative studies reported improvements in anxiety, mood, ED symptoms and increased food eaten and weight gain. However, robust conclusions were limited by small sample sizes, a lack of control group, non-randomisation, or another intervention being implemented with the MBI. Overall, reporting of MBI components was inconsistent, with a mean completeness of 45–100%.

**Discussion:**

To our knowledge, this is the largest scoping review to map the breadth and depth of studies assessing MBIs in ED treatment. Preliminary evidence supports positive psychological and behavioural outcomes for patients with EDs. However, conclusions are limited by lack of methodological rigor, variable outcome measures, and inconsistent reporting of intervention components or theoretical framework. The complex and multifaceted nature of EDs and high rates of comorbidities and trauma histories further complicates interpretations. To advance research and clinical application of MBIs in ED care, standardized approaches to intervention design and reporting are needed, as well as randomised controlled studies clearly testing MBIs against other interventions.

## Introduction

Individuals diagnosed with eating disorders (EDs) present with a variety of eating-related behaviours that negatively impact nutritional intake, affect psychological and psychosocial wellbeing, and result in somatic complications (1, 2). ED diagnoses (including anorexia nervosa, bulimia nervosa, binge eating disorder and other specified feeding or eating disorders) have an onset at the developmentally vulnerable period of adolescence (ages 15–25 years), with a peak age onset of 15.5 years and an average duration of six years (3, 4). Further, mortality rates for individuals with EDs are higher than the general population: a six times higher mortality rate for those with anorexia nervosa (AN), and a doubled mortality rate for those with other EDs (5).

Research indicates significant increases in EDs over the past two decades (6–8). An estimated 55.5 million people globally were living with an ED in 2019 (9). In 2020, during the COVID-19 pandemic, there was a reported 15% increase in the incidence of EDs (10, 11). Overall, the data indicate a worsening burden of the illness (7) and an increase in the associated treatment and economic costs due to an increasing prevalence of EDs (12).

The complex profile of ED patients is exacerbated by multiple comorbidities, with an estimated 56%-95% presenting with an additional psychiatric disorder, depending on the type of ED (13–15). The most prevalent comorbidities are substance use disorders (SUD), ranging from 20% to 51% (16), followed by post-traumatic stress disorder (PTSD) at 25% (17). Further, 33% of individuals with EDs have experienced sexual trauma and 21% physical assault (18, 19). Perfectionism, and impaired affect regulation and cognitive processing are common features in EDs which complicate treatment (20, 21). Additionally, there are a myriad of medical complications associated with EDs because of malnutrition, purging, laxative abuse, excessive exercise, and weight loss (22, 23). Thus, ED behaviours can impact every system of the body, resulting in gastrointestinal, dermatologic, endocrine, neurological, haematological, pulmonary, and cardiac issues and abnormalities (24–29).

ED treatment programs apply psychological therapies, which address different presenting issues common to individuals with EDs (30). Therapies include cognitive behavioural therapy (CBT), dialectical behaviour therapy (DBT), family-based therapy (FBT), and interpersonal therapy (IPT) (31). The focus of CBT is on addressing thoughts and feelings as these relate to and impact ED behaviours (31). DBT addresses issues related to affect regulation and how this impacts ED behaviours (32, 33). FBT focuses on engaging families as a resource to help make changes to reduce and eliminate ED behaviours (34). IPT aims at addressing interpersonal issues and how disordered eating and ED behaviours impact relationships (35). However, estimates indicate that ED treatment is effective for about 50% of individuals (36), suggesting that ED treatment needs further development to more adequately address the complex matrix of ED behaviours (37, 38).

### Music-based interventions in eating disorder treatment

Music-based interventions (MBIs) include a broad range of practices that utilise music to support health and wellbeing. These can integrate the use of music in various ways, including listening to, singing, playing, composing, and improvising music (39). MBIs can be distinguished by the way one engages with the music (for example, listening to songs and discussing them, listening to instrumental music for relaxation, actively playing instruments or singing songs, improvising instrumentally or vocally, composing a song or instrumental music) and by who facilitates the MBI. MBIs may be facilitated by qualified music therapists (music therapy) or other healthcare professionals (non-music therapists) as well as musical experiences led by community musicians or the use of self-selected music implemented by the client themselves (39). The accessible nature and myriad ways of engaging with music allows for the use of MBIs in various contexts and for a range of client needs, across therapeutic and community-based settings (27). While music therapy is encompassed in this broad category, it is a specialist-delivered intervention differentiated by formal training and qualification. Music therapists are allied health professionals trained in a wide range of music therapy theories, approaches and methods to address therapeutic goals across clinical populations. Music therapists use both active and receptive methods, adapting sessions reflexively to meet emerging and changing needs of the individual or group throughout the therapeutic process (40–42). MBIs therefore can encompass a continuum of music use for wellbeing, from personal use to specialist music therapy sessions (43).

There has been a recent increase in reviews evaluating the use and potential outcomes of music in people with or at risk of EDs (44–47). Testa et al. completed a systematic review that included 16 studies examining the effects of music in people with EDs or at risk of EDs. Studies were limited to experimental or observational designs which reported measurable outcomes, and thus only one qualitative study was included (44). Results indicated that listening to music improved nutritional intake, group singing reduced anxiety after meals, while songwriting assisted in processing various therapeutic issues. A recent systematic review of controlled studies utilizing music therapy in ED treatment found no randomised studies and only two relevant publications from one treatment-as-usual (TAU) controlled study (45). Pre-post results (quantitative and qualitative) indicated a decrease in post-meal stress and anxiety following 1-hour music therapy group sessions twice per week, which was greater for the music therapy intervention than TAU (48, 49). The group sessions integrated various music therapy methods such as singing, listening to music, and songwriting. Chang et al. conducted a narrative systematic review that evaluated therapeutic outcomes in individuals with EDs, where music was a component of the intervention (46). Results from 16 quantitative, qualitative and mixed methods studies indicated that the intervention improved mood regulation, helped manage distress associated with mealtimes, and improved emotional wellbeing. The authors indicate generalisability of the results is limited due to small sample sizes (16 studies ranged from 3 to 20 participants) and the lack of detailed information about the MBIs. While in a recent systematic review exploring the extent to which music therapy and systemic or family-based therapy is integrated into ED treatment, the examination of 26 studies revealed common parallels across where both facilitated embodiment and interconnectedness, served as a gateway of deeper engagement with self and illness, fostered and self-expression, and supported emotion regulation (47).

Each of these systematic reviews have captured a corpus of the literature, helping to explicate the use of music in the context of EDs or subclinical disorder. However, there has been a lack of singular focus on studies where music is a key component to the intervention, which is instrumental to a clearer understanding of the impact of MBIs. For example, all the above reviews except one (45) included studies where background music was played in a video, but music was not a focus of the intervention. Further, systematic reviews can limit the type of research literature included, resulting in rich therapeutic descriptions from clinical case studies being excluded. A recent study revealed widespread adaptation of music therapy methods to meet the needs of patients in ED treatment (50). Case studies illustrating these varied and nuanced ways of delivering and engaging with MBIs would further inform literature reviews and support clinicians employing MBIs in building an evidence-based practice.

Therefore, the aim of this scoping review was to encompass studies where music comprised an essential component of the intervention in a therapeutic ED setting, including case studies to contextualise and more comprehensively understand the use of MBIs in the context of ED treatment. A challenge in reviewing and evaluating MBIs in research is that there is often a lack of clarity in the literature due to inconsistent terminology and quality of reporting (51, 52). As a result, MBI reporting guidelines were introduced in an effort to improve the transparency and specificity of MBIs as well as to evaluate the quality of the research and ensure that it can be replicated (51). The present scoping review therefore also expands on prior systematic reviews by examining the clarity of reporting based on these MBI reporting guidelines.

## Methods

A scoping review of the literature was conducted, using a systematic and iterative approach to evidence synthesis. This adhered to the Preferred Reporting Items for Systematic Reviews and Meta-Analyses extension for Scoping Reviews (PRISMA-ScR) guidelines (53) and was pre-registered with the Open Science Framework (https://osf.io/4xv9f).

### Search strategy

Eight electronic databases were queried for relevant articles from inception to May 2025, including MEDLINE (National Library of Medicine), Embase (Elsevier), Scopus (Elsevier), APA PsycArticles (American Psychological Association), APA PsycINFO (American Psychological Association), Psychology and Behavioural Sciences Collection (EBSCO), Web of Science (Clarivate), and Cochrane Library (The Cochrane Collaboration). The following search terms were applied: (music therapy OR music-based intervention OR guided imagery and music OR songwriting OR song composition OR drumming OR music listening OR music improvisation OR song discussion OR active music making) AND (eating disorders OR anorexia nervosa OR bulimia nervosa OR binge eating disorder OR pica OR rumination disorder OR avoidant restrictive food intake disorder OR unspecified feeding OR eating disorder). There were no restrictions on publication date or status; however, articles were filtered by subject (humans only), study type (peer-reviewed), and language (English). In addition to database searches, reference lists and relevant conference abstracts were hand-searched to identify supplementary eligible studies.

### Study selection

The following inclusion criteria were applied to evaluate eligibility in the qualitative synthesis: (1) patients of any age and sex with a primary ED diagnosis; (2) MBIs (i.e. any intervention utilising music as a core component, including music therapy and other non-music therapist delivered music experiences used in the treatment or management of EDs across any clinical or community setting; (3) qualitative, quantitative, and mixed methods studies, whether prospective or retrospective, including case reports; and (4) peer-reviewed articles and published in the English language. To ensure breadth and relevance, inclusion was not limited by study design, publication status, or geographical location. Primary outcomes of interest encompassed physical and mental health following the implementation of MBIs. We included all study designs, excluding review articles, book chapters, nonhuman studies, conference abstracts, and articles in a language other than English. Studies were excluded if ED was not the primary diagnosis, if the MBI was not a key component of the intervention, or the paper was not available in English. Screening of the articles was independently performed by four reviewers (AH, AR, JB, JG). Any disagreements were resolved during discussions between the reviewers.

### Data charting

Data from eligible studies were charted by five independent reviewers (AH, AR, JB, JG, TM), including two (JG, TM) with no prior content knowledge of the topic. This approach allowed for both subject-matter expertise and naïve interpretation, helping to minimize potential bias while enhancing objectivity and transparency. Using a standardised charting form created in Microsoft Excel, the following variables were extracted: author, publication year, country, study design, and outcome measures; sample size, participant age, and ED diagnosis; intervention type, duration, and setting; and primary outcomes and any other pertinent findings. Details were recorded when studies indicated the facilitator’s theoretical approach and methods or techniques used. A sixth reviewer (SASN) examined all extracted data to ensure alignment with inclusion criteria and internal consistency. Discrepancies were resolved through team consensus. The charting form was updated iteratively to accommodate emerging themes and variations in data reporting across studies.

### Evidence synthesis

Qualitative and quantitative studies were summarised in a narrative format. Qualitative data were then analysed thematically. Additionally, evaluation of MBI reporting for each study was also conducted following established MBI reporting guidelines (51, 54).

### Qualitative data analysis

Qualitative data was integral to 17 of the studies and was initially reviewed by one research team member (AH) to begin the reflexive thematic analysis. This process entailed reviewing and coding the data following the six-step process (55, 56). Emergent themes were identified based on integrating the outcome data from these studies. This iterative process included grouping themes into categories of similar orientation. The next step involved examining these categories to identify points of connection and patterns. This led to developing subordinate themes which were then reviewed by all members of the research team and integrated into a comprehensive framework. When discrepancies emerged, the reviewers returned to the data to inform their discussion and understanding of the themes. This iterative process continued until reviewers (AH, SN) reached a mutually agreeable decision. Following this, other research team members were asked to complete a final review of the themes to ensure full team consensus.

## Results

**Figure 1:**
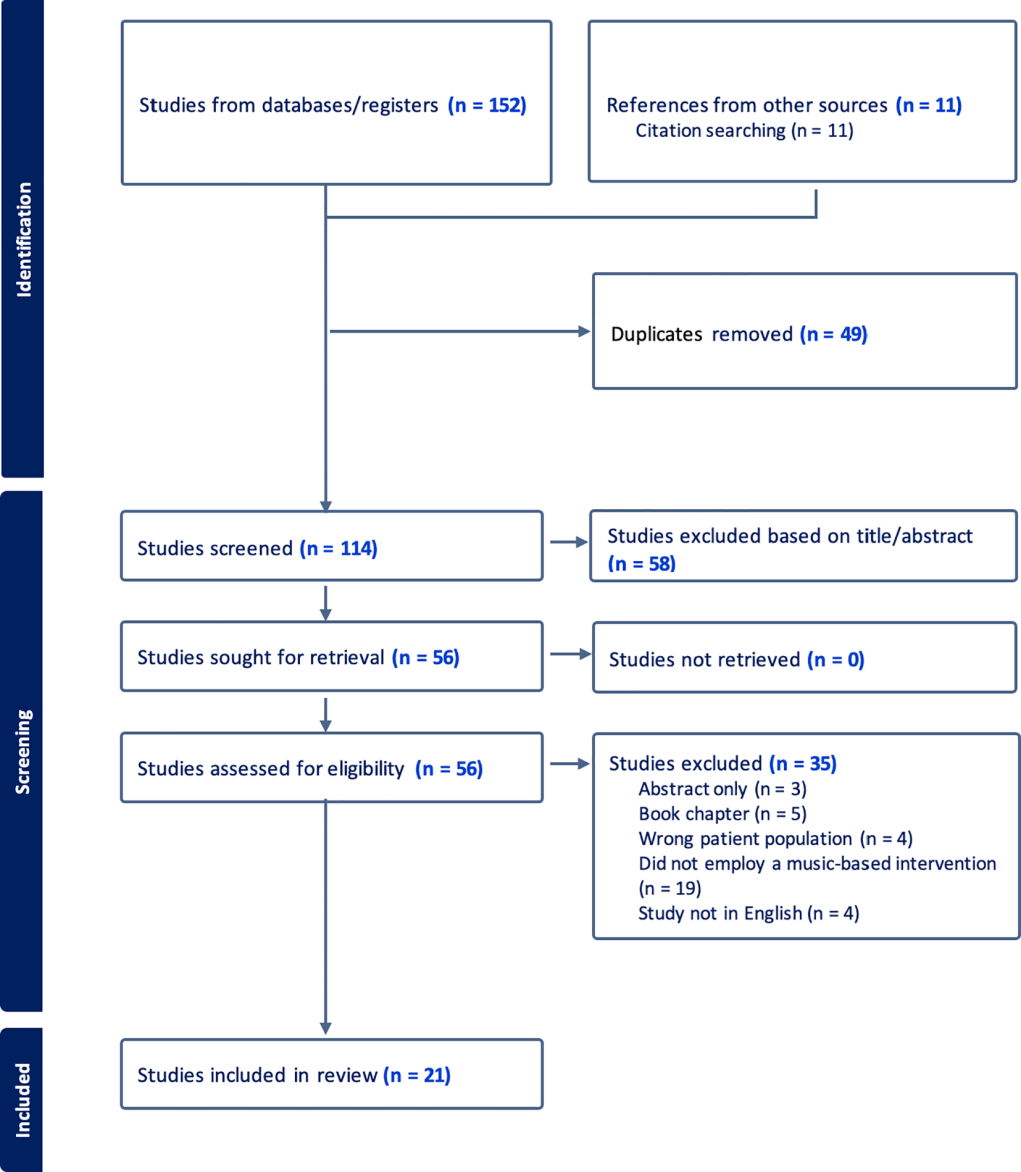
Flowchart of search process.

The literature search identified 163 relevant citations. After removing duplicates and conducting title and abstract screening, 56 articles remained for further evaluation. Full texts of all potentially eligible papers underwent a thorough review, resulting in the exclusion of 35 articles. After careful consideration, 21 articles met all the criteria and were included in the scoping review. The PRISMA flow diagram illustrating the study selection process can be seen in **Figure 1**.

### Study characteristics

Study characteristics and participant demographics are reported in **Table 1** (qualitative studies) and **Table 2** (quantitative studies) including country of publication, study design, sample size, participants’ age, gender, participant diagnostic information, type of music intervention, source of outcome assessment used, setting, and assessment based on reporting guidelines. This included identifying how many of the 11 recommended and relevant components from the 7 criteria were included in the MBI reporting of the study (50, 53). The component of treatment fidelity was not included in evaluating case reports as it is not relevant to this type of research.

**Table 1:** General characteristics of qualitative studies.

Author, year	Country	Study design	Total N	Age	Female %	Eating disorder^1^	MBI	Outcomes	Setting	Reporting guidelines
Bauer, 2010 (57)	Germany	Case study (pre-post)	1	28	100	Bulimia nervosa	Individual music therapy (Instrumental improvisation)	Therapist observation	Inpatient	100%
Fendel et al., 2018 (58)	Germany	Qualitative interviews post-intervention	20	M:28.3	100	Anorexia nervosa^1^	Individual vibro-acoustic stimulation	Subjective bodily experiences during treatment explored through semi-structured interviews	Inpatient	45%
Heal and Hara, 1993 (59)	UK	Case study	1	28	100	Anorexia nervosa^1^	Individual music therapy (Instrumental improvisation and song composition)	Therapist observation	Outpatient	91%
Heiderscheit and Madson, 2015 (60)	US	Case study	1	57	100	Eating disorder not otherwise specified^1^	Individual music psychotherapy (Song/music collage/playlist)	Self-reported measures of depression (10-Point Likert Scale), anecdotal reports and clinical observations	Outpatient	91%
Heiderscheit and Murphy, 2021 (61)	US	Case study	1	27	100	Anorexia nervosa^1^	Individual music therapy (Music relaxation, directed music imaging, stimulative music listening, toning, and chanting)	Anecdotal reports and clinical observations	Outpatient	91%
Heiderscheit, 2023 (62)	US	Qualitative	8	M:36 R:23-58	100	Anorexia nervosa, bulimia nervosa, binge eating disorder, other specified feeding or eating disorder^1^	Individual music psychotherapy (Bonny Method of Guided Imagery)	Self-reported measures of feasibility and helpfulness, thematic analysis of feedback	Both inpatient and outpatient	100%
Heiderscheit, 2024 (63)	US	Qualitative	8	M:36 R:23-58	100	Anorexia nervosa, bulimia nervosa, binge eating disorder, other specified feeding or eating disorder^1^	Individual music psychotherapy (Bonny Method of Guided Imagery)	Thematic and intertextual analyses of GIM session transcripts	Both inpatient and outpatient	100%
Hilliard, 2001 (64)	US	Case study	NR	R:14-55	NR	Eating disorder not otherwise specified^1^	Group & individual music therapy (songwriting, singing, drumming, and song discussion)	Anecdotal reports and clinical observations	Inpatient	82%
Lejonclou and Trondalen, 2009 (65)	Sweden	Case study	2	R:19-35	100	Anorexia nervosa, bulimia nervosa	Individual music therapy (Song discussion, Instrumental improvisation, song composition, singing and music and movement)	Descriptions of the two patients’ experiences, observations, drawings, and writings; therapist observation	Inpatient	82%
McFerran et al., 2006 (66)	Australia	Qualitative (analysis of patients’ song lyrics)	15	M:15 R:12-17	100	Anorexia nervosa	Individual music therapy (songwriting)	Lyric analysis - a modified content analysis approach to categorise the lyrics from songs written by the patients into different themes	Inpatient	64%
McFerran et al., 2008 (67)	Australia	Qualitative (analysis of patients’ song lyrics)	15	M:15 R:12-17	100	Anorexia nervosa	Individual music therapy (songwriting)	Lyric analysis - a modified content analysis approach to categorise the lyrics from songs written by the patients into different themes	Inpatient	64%
Pavlakou, 2009 (68)	UK	Qualitative	8	R:18-62	100	Bulimia nervosa, anorexia nervosa, binge eating, chronic dieter, emotional eating	Group singing workshop	Descriptions of the patients' experiences, diaries, thematic analysis of feedback	Outpatient	82%
Robarts and Sloboda, 1994 (69)	UK	Case study	2	M:15.5R:11-20	100	Anorexia nervosa	Individual music therapy (Instrumental improvisation)	Therapist observation	Inpatient	91%
Robarts, 2000 (70)	UK	Case study	1	14	100	Anorexia nervosa^1^	Individual music therapy (Instrumental improvisation)	Therapist observation	Inpatient	91%
Shah et al., 2021 (71)	Canada	Mixed-methods design (pre-post and focus groups as well as verbal and music contributions in music therapy)	21	R:16-58	95	Anorexia nervosa, bulimia nervosa^1^	Group music therapy (Instrumental improvisation, singing, drumming, music listening)	Self-reported measures of Positive and negative affect scale, Difficulties in emotion regulation scale, Emotion regulation questionnaire, therapist observation, transcripts from focus groups	Inpatient	73%
Trondalen and Skårderud, 2007 (72)	Norway	Case study	1	19	0	Anorexia nervosa	Individual music therapy (Instrumental improvisation)	Musical improvisation sessions recorded, semi-structured interview after therapy, therapist's reflective notes	Outpatient	91%
Trondalen, 2003 (73)	Norway	Case study	1	26	100	Anorexia nervosa	Individual music therapy (Instrumental improvisation and music listening)	Therapist observation	Outpatient	91%

^1^Indicates studies that reported comorbid mental health diagnoses which included Bipolar Disorder, Borderline Personality Disorder, Dissociative Identity Disorder, Generalized Anxiety Disorder, Major Depressive Disorder, Obsessive Compulsive Disorder, and Post-traumatic Stress Disorder, Substance Use Disorder. M, mean; R, range.

**Table 2:** General characteristics of quantitative studies.

Author, Year	Country	Study design	Total N	Age	Female %	Eating disorder^1^	MBI treatment intervention	Outcome assessment	Setting	Reporting guidelines
Bibb et al., 2015 (48)	Australia	Non- randomized pre-post design, pilot study	18	R: 20-58	94	Anorexia nervosa	Group music therapy (Singing, song discussion, and song composition)	Self-reported measure of subjective units of distress scale (anxiety/distress)	Inpatient	64%
Bibb et al., 2019 (49)	Australia	Single group pre-post design	13	R:18-41	100	Anorexia nervosa^1^	Group music therapy (Singing and song discussion)	Self-reported measure of Subjective units of distress scale (anxiety/distress)	Outpatient	82%
Meneguzzo et al., 2024 (74)	Italy	Randomised within-subjects design	51	M:25.22	100	Anorexia nervosa, bulimia nervosa, binge eating disorder, other specified feeding and eating disorders	Groups included three different background music conditions during lunch and dinner. Conditions included no background music, continuous classical music featuring only a piano, and a pop music playlist. All participants participated in ten meals, being exposed to all conditions at least three times, with condition order randomised.	Self-reported scales: Positive and Negative Affect Schedule, measures of hunger, satiety, desire to eat, perceived stress, and mealtime difficulty, music volume, loudness, and its pleasantness post-meal. Observations by trained dietitians: presence of eating rituals, adherence to mealtime duration, food consumption.	Inpatient	45%
Wang and Xiao, 2021 (75)	China	RCT, pre-post design, per-protocol analysis excluded dropouts	66	M:16.53 R:14-19	92	Anorexia nervosa	Music therapy combined with cognitive behavioral therapy (Music listening) Unspecified whether individual or group sessions	Self-reported measures of eating disorder examination questionnaire, Beck anxiety inventory, Beck depression inventory, treatment satisfaction, Body mass index, and abdominal subcutaneous fat thickness measured by the therapist	Inpatient	55%

^1^Indicates studies that reported comorbid mental health diagnoses which included Bipolar Disorder, Borderline Personality Disorder, Dissociative Identity Disorder, Generalized Anxiety Disorder, Major Depressive Disorder, Obsessive Compulsive Disorder, and Post-traumatic Stress Disorder, Substance Use Disorder. M, mean; R, range.

The majority of included studies were carried out in Europe (n=10) followed by North America (n=6), Australia (n=4), Asia (n=1), for studies with available data. Overall, 254 participants (range:1-66) were included from ages 11 to 62 for studies with available data. The research methods used were primarily qualitative (n=16), with the remainder quantitative (n=4) or mixed methods (n=1). Most studies reported on anorexia nervosa (n=19), followed by bulimia nervosa (n=7), binge eating disorder (n=4), other specified feeding or eating disorder (n=4), emotional eating (n=1), chronic dieter (n=1), and eating disorder not otherwise specified (n=4). Evaluation of reporting guidelines differed across studies, with a range of 45-100% and a mean of 76%, equivalent to reporting on nearly 8 of the 11 components.

MBIs were identified when details regarding the music experience were provided. MBIs are reported as music therapy when facilitated by a qualified music therapist and other MBIs (non-music therapist delivered) are reported by the type of intervention only. Within the included studies, the majority investigated music therapy (n=18). Music therapy sessions included individual (n=12), group (n=4), and both individual and group sessions (n=1). One study did not specify whether the sessions were in a group or individual format. Music therapy sessions utilized receptive music experiences (n=10) (song discussion, music listening for relaxation, or music directed imaging), instrumental improvisation (n=8), songwriting or song/music collage (n=7), singing or chanting/toning (n=5), drumming (n=2) or movement to music (n=1). One music therapy intervention combined music listening with cognitive behavioural therapy. MBIs led by non-music therapists (n=3), two qualitative and one quantitative) included vibroacoustic stimulation, a singing workshop, and music listening during mealtimes. Details of the music therapy theoretical approaches, when indicated, are further outlined in subsequent sections.

### Qualitative results

Qualitative studies included a total of 106 participants, which were comprised predominantly of case reports/studies (n=10), and qualitative designs (n=6), followed by mixed methods pre-post design (n=1). Nine were conducted in an inpatient setting, six in outpatient settings, and two studies across both inpatient and outpatient settings. Gender of study participants was between 95-100% female; one case report did not report gender. Nine of the studies reported comorbid mental health diagnoses that included bipolar disorder, dissociative identity disorder, generalized anxiety disorder, major depressive disorder, obsessive compulsive disorder, post-traumatic stress disorder, and substance use disorder. Study outcomes were based on therapist observations (n=9), self-report (n=3), analysis of content from sessions (song lyrics, musical improvisation, imagery) (n=5), and semi-structured interviews (n=2). Evaluation of the reporting of the qualitative studies represents a range of 45-100% with a mean of 84%, reflecting reporting of approximately 9 of the 11 components.

**Table 3** highlights study duration within the context of design, detailing information on the MBI, theoretical approaches, and any other intervention components. Finally, the main results from the studies are indicated. We note that length of intervention varied widely across these studies, from two weeks to 2.5 years. Main results were further explored through the thematic analysis. This analysis resulted in seven emergent outcome themes being identified including: building interpersonal capacity, managing the emotional landscape, developing capacity in self, embracing novelty and growth, mental processes, experience and relationship with body, and fostering engagement in ED treatment. **Table 4** presents the 7 themes with the 13 emergent subthemes, along with related outcomes and quotes. Quotes from the studies included in the analysis provide examples of content that informed the themes. We note that negative outcomes were only reported in one study and consisted of experiencing the body more consciously and a negative evaluation of new experiences.

**Table 3:** Results of the qualitative studies.

Author, year	Study duration and design	MBI	Other interventions received^1^	Main outcomes
Bauer, 2010 (57)	10-months, case report (pre-post)	Music therapy	None	Improved self-awareness, emotional expression and sense of community as well as mutual support, increased motivation.
Fendel et al., 2018 (58)	2-weeks, qualitative interviews post-intervention	Three one-to-one vibro-acoustic stimulation sessions with the Body Monochord (“BoMo”, body sound treatment instrument)	The standard treatment for eating disorders	Qualitative analysis of interviews using grounded theory found the following categories related to the subjective bodily experiences of participants during the BoMo sessions: Differentiated perception; Focused attention; Emergence of body-related feelings; Emergence of emotions; Emergence of thoughts; Emergence of inner images; Relaxation; Spatial and temporal experience; New bodily experiences; Self-reflection Experiences during treatment were not exclusively focused on body problem areas and were independent of patient body shape or weight and had both positive and negative connotations.
Heal and Hara, 1993 (59)	approx. 1.5 years, case study	Music therapy (improvisation or spontaneous sound pictures)	Weekly psychotherapy sessions over a year	The clinic reported changes: Client was able to report her ideas and thoughts verbally (no longer needing to write anything down) and was able to verbalise her concerns, activities, and anxieties or unhappiness. Her weight remained stable although she reported that she did have episodes of anorexia nervosa behaviours when stressed.
Heiderscheit and Madson, 2015 (60)	4-weeks, case report	Iso principle: music psychotherapy using systematically programmed classical music (therapeutic playlist) to change and manage different mood states and tempos	None	The patient’s depression worsened mid-treatment prompting switch from the Bonny Method of Guided Imagery in Music to iso principal method; increased hope, optimism, and empowerment; greater ability to manage depression and engage in social activities; improved engagement in treatment and recovery.
Heiderscheit and Murphy, 2021 (61)	3-months, case report	Trauma-informed music therapy (co-created, adapted, and tailored to patient’s strengths, interests, and music preferences)	Daily practices of coping skills	Shame reported when using symptoms vs. new coping skills; responded better to stress and anxiety by end of treatment; learned to relinquish maladaptive skills and interrupt pathological cycles; improved coping behaviours and emotion dysregulation allowing for continued trauma work.
Heiderscheit, 2023 (62)	12-months, qualitative	Bonny method of guided imagery: in-depth music psychotherapy using therapist-selected classical music to elicit emotional processing	Optional homework post-sessions, journal or create mandala	Therapist observation throughout sessions and post study questionnaires showed: initial apprehension; perceived benefit: insight, emotional processes, and growth; perceived challenges: fear of unfamiliar and learning to trust self; no disruption to treatment schedule or program; empowered participants to engage in treatment planning and recovery process; highly feasible to implement across levels of treatment.
Heiderscheit, 2024 (63)	12-months, qualitative	Bonny method of guided imagery: in-depth music psychotherapy using therapist-selected classical music to elicit emotional processing	Optional homework post-sessions, journal or create mandala	Thematic analysis of imagery themes from 116 GIM session transcripts. Three themes and nine sub themes emerged. including emotional landscape (feeling stuck, acknowledging emotions, working through unresolved emotions), relationships (self, others and ED), and experiencing transformation. Intertextual analysis indicated a therapeutic arc representative of The Hero’s Journey
Hilliard, 2001 (64)	NR, qualitative	Cognitive-behavioural music therapy	None	Therapist observation throughout sessions: stress and fatigue reported during behavioural and cognitive stages, respectively; decreased stress, anxiety, and discomfort, especially during and after meals; improved coping skills, empowerment, and laughter; group sessions were well-attended and well-received by patients, families, and professionals.
Lejonclou and Trondalen, 2009 (65)	1st case - 3 years; 2nd case - 2 years, clinical case study	Individual music therapy using a psychodynamic approach, focusing on relating experiences through music (songwriting, improvisation, movement/dance, listening to music, and verbal processing)	1st case - training activities of daily living. 2nd case - none	1st case – Participant gained weight, left the hospital and returned to a normal life. Participant acquired a new self- confidence and linked positive feelings to her body. 2nd case – The participant became more satisfied with her body. The binging and purging almost disappeared after the therapy. Participant was able to share ‘parts of her feelings’ and talk about bulimia nervosa diagnosis with some of her friends.
McFerran et al., 2006 (66)	2-years, qualitative (analysis of patients’ song lyrics)	Individual songwriting sessions	Multidisciplinary eating disorder program	The theme of ‘identity’ was used most frequently (28%) in the lyrics, with the sub-theme of ‘exploring new behaviours, positive self-talk’ being addressed most often (12.5% of total). Songwriting was able to reveal information that had not been discussed with other treatment team members.
McFerran et al., 2008 (67)	2.5-years, qualitative (analysis of patients’ song lyrics)	Individual songwriting sessions	Multidisciplinary eating disorder program	The sub-theme ‘relationship with mother’ was used 8.15% in the lyrics. The results highlight the importance of the mother-daughter relationship for adolescents with eating disorders. Descriptive results show an avoidance of negativity and an emphasis on the positive and not placing blame on parents.
Pavlakou, 2009 (68)	3-weeks, qualitative	Group singing workshop	Physical warm-ups, stretching, breathing and vocal exercises at the beginning of every session	Through the singing activity, participants reported physical relaxation, awareness of their body, emotional release and ability to distance themselves from their everyday problems. Through the rehearsal process, participants reported increased self-esteem, opportunities to express themselves freely, and were able to value and appreciate themselves, as well as boost of confidence, and a sense of accomplishment. Through the group experience, participants reported healthy interactions with group members (meeting new people, socialising and interacting with group members during sessions and the breaks); feeling connected with the group members; sense of belonging; feeling of equality due to issues with food; Overall, the group workshops helped participants to feel less stressful, more empowered and positive, and to increase their self-esteem.
Robarts and Sloboda, 1994 (69)	4-months, case study	Music improvisation (instrument playing)	Ward treatment and re-feeding programme	1st case- The patient left the unit and did not require readmission. The therapist considered the music played in the last session to be much more interactive than the earlier playing. The music had a sense of wholeness and integration. The therapist described the music as 'folksy', 'earthy' and 'grounded'. The patient also initiated musical ideas and responded to the therapist ideas. The therapist was left with a sense of a personality that is strong and alive. 2nd case- The patient left the unit after nine months and did not require readmission. Positive feedback from a therapist. The patient began to develop an acceptance of herself and began to overcome her defensive as well as controlling behaviour. The playing became more spontaneous, self-expressive and exploratory.
Robarts, 2010 (70)	NR, case study	Music therapy	Inpatient care	Case material used to illustrate the clinical function and application of musical phenomena, in particular the “tonal-rhythmic field of sympathetic resonance” and the use of motif.
Shah et al., 2023 (71)	4-weeks, pre-post design, mixed methods quantitative results reported in **Table 5**	Group music therapy sessions using a combination of improvisation, singing, drumming, listening back to recorded group improvisations and responding through visual art, and mindfulness-based exercises	Inpatient eating disorder treatment - largely group-based, including psychoeducation and psychotherapy sessions based on dialectical behaviour and cognitive behavioural therapy, creative arts programming, recreative and horticultural therapies	Positive and negative affect scale showed that participants experienced a decrease in negative affect (p = 0.006) after 4-week music therapy programme, compared to pre-intervention data. There was no significant overall change in positive affect. Qualitative data suggested that participants discovered music’s ability to represent various aspects of themselves and their recovery journeys, music’s potential to support them to externalise, shift, and stay with emotions, and music’s capacity to foster social connection.
Trondalen and Skårderud, 2010 (72)	12-months, case report	Individual music therapy sessions involving free improvisation	None	High participation rate of 83% when invited to write songs. Songwriting allowed exploration of the theme of identity development. Musical improvisation facilitated "affect attunement" - sharing/matching of inner feeling states through intensity, form, timing, etc. Suggested a link between the musical relating experience and subsequent verbal processing could support a more coherent sense of self.
Trondalen, 2003 (73)	10-months, case report	Music therapy involving improvisation, singing, instrument playing, “self-listening" experience	Psychotherapy	The "self-listening" experience became very important in the therapeutic process. The experience promoted a sense of belonging in time and space.

^1^no control conditions were present in any of the qualitative studies

**Table 4:** Qualitative Studies: Outcome Themes following MBIs.

Themes	Sub themes	Outcomes	Quotes
Building interpersonal capacity	Promoting social engagement Fostering a sense of belonging	Fostered social engagement and connection Supported affect attunement Supported exploration of relationships Fostered development of healthy relationships Reduced feelings of isolation Decreased feelings of loneliness Musicking provided experience of support	“Engaging in making music with others provided a way to connect” “Making music with others fostered healthy social interactions” “Co-creating music offered a shared emotional experience” ‘’Engaging with others in music helped foster recognition of the need to connect” “Offered positive and shared creative experience” “Making and sharing music fostered sense of support”
Managing the Emotional Landscape	Fostering emotional expression Coping with emotions Managing stress	Developed willingness to explore feelings Increased willingness and capacity to express emotions Helped shift emotions Helped to cope with and process difficult emotions Elevated and managed mood Fostered physical relaxation Provided opportunities to practice stress management Decreased stress	“Making music (nonverbal) serves as a referent to project, explore or recast one’s inner experiences.” “Music experiences afforded opportunities to practice feeling and tolerating difficult emotions.” “Listening to and creating music provides a safe way to externalize and explore emotions.” “Listening to and making music provided a way to shift and change mood.” “Using music as a resource to manage stress and anxiety provided a comfortable way to engage one’s body physically.” “Music therapy decreased the different intensities of meal-related stress and anxiety.” “Music therapy helped significantly reduce anxiety at different stages of recovery.”
Developing Self-capacity	Insight Self-esteem	Fostered self-reflection Fostered development self-awareness Fostered change in self-perception Improved self-appreciation Fostered positive self-talk	“Fostered a fusion of personal narratives to challenge and change perception.” “Music experiences fostered exploration and externalization of inner experiences to help make meaning and comprehend.” “Engaging in music experiences enables an exploration of one’s identity beyond that of the ED (discovering their musical identity).” “Creating music (songwriting) provided a means to practice sharing personal information and communicating positive messages to oneself.”
Embracing Novelty and Growth	Engagement with novelty Personal development	Fostered development of spontaneity Supported exploration of new behaviors and skills Provided opportunities to practice new skills Fostered change in personal growth Supported exploration of aspects of self Perspective and insight gained on one’s life story Fostered hope for future & recovery	“Creating and making music provided opportunities to engage in new experiences.” “Leaning into new ways of engaging with music fostered facing fears.” “Engagement with symbolism and metaphor in music experiences supported engagement in cognitive and affective processes that support growth and transformation.” “Embodied aesthetic experience allows an individual to project their unique feelings, thoughts, and experiences to be explored, and they can exert psychological control and pace their process.”
Improving Mental Processes	Cognition	Served as a means of cognitive distraction Decreased defenses and controlling behaviors Improved focused attention	“Music provided a means of distraction to manage anxiety and intrusive thoughts.” “Engaging in making music provides opportunities to be present in the moment and not focused on ED.”
Improved Relationship with Body	Body awareness & comfort	Provided new bodily experiences Fostered awareness of body (spatial and temporal) Improved self-image Decreased discomfort during and after meals	“Engaging in music afforded experiences to start to move into one’s body.” “Active music engagement supports developing a closer connection with the mind and body.” “Self-exploration in music and imagery experiences enables individuals to see themselves from new and different perspectives.” “Music therapy as a distress tolerance technique (at mealtime) emphasises practicing through experience rather than discussion.”
Engagement in ED treatment	Motivation Revealing unaddressed issues	Demonstrated high rate of participation Well received by patients and families Improved engagement in treatment Improved motivation for recovery Identified issues not explored by other members of treatment team Uncovered issues unaddressed in verbal therapy	“Music therapy clinicians report that groups are well attended.” “Patients and their families share with staff how music therapy has helped in crisis, challenged cognitive distortions, and helped them gain insight.” “GIM session fostered discovery of inner resources vital to moving forward in recovery.” "Music therapy enabled exploration and expression of complex feelings and experiences that were difficult to address in verbal therapy.” “GIM supported exploration of therapeutic issues unresolved or unaddressed in verbal therapy.”

**Figure 2:**
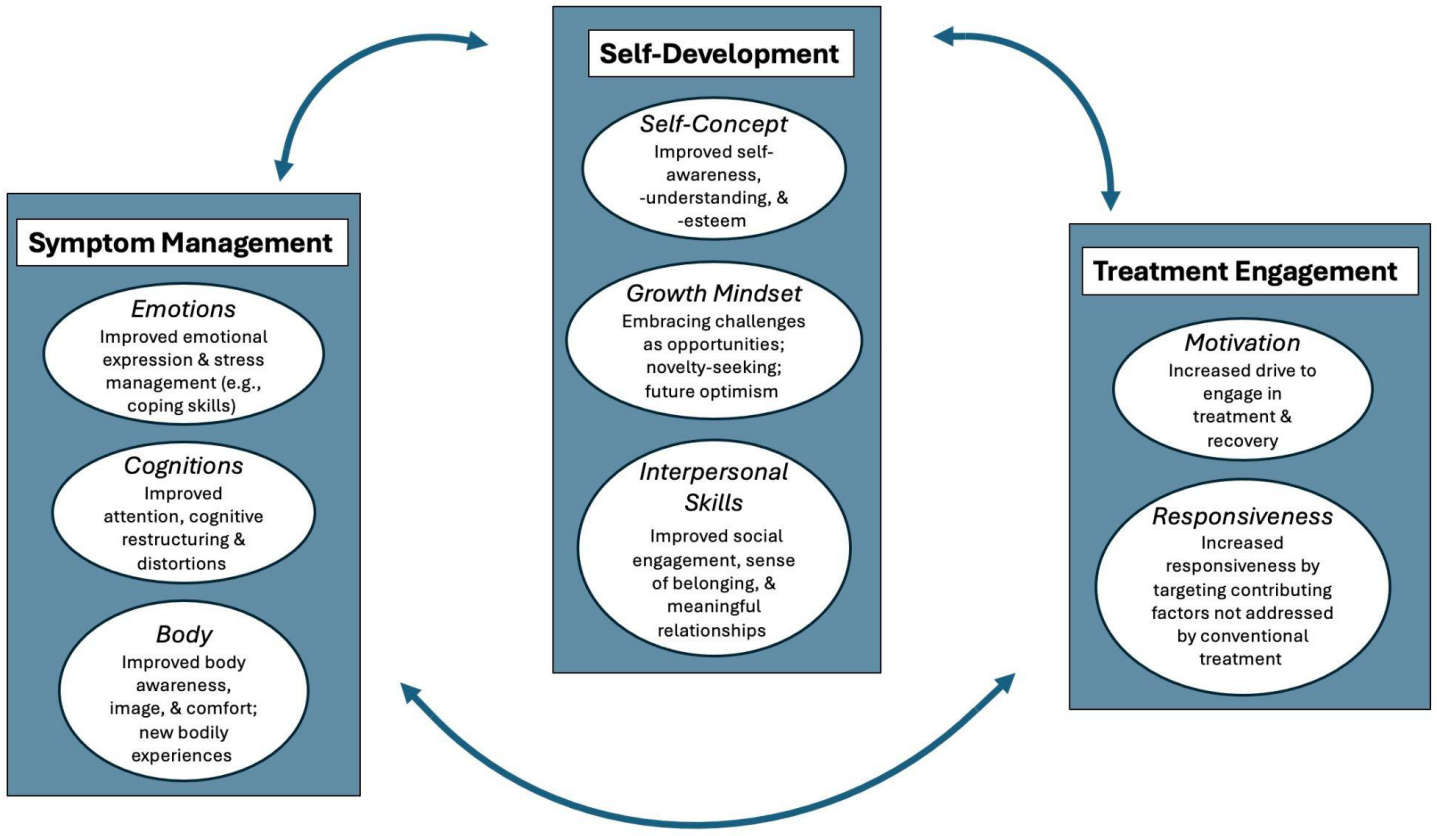
Alignment of emerging qualitative outcomes with ED treatment and recovery processes.

Organization of these themes reflects various stages of the treatment and recovery process. **Figure 2** illustrates how these themes follow differing levels of treatment, beginning with symptom management that focuses on fostering emotional expression, stress management and developing coping skills which leads to improved attention and cognitive restructuring, leading to improved body awareness and body image. This therapeutic work supports transitioning to self-development that highlights improved self-awareness and self-esteem, developing a growth mindset through engaging in new and novel experiences, and supporting the development of interpersonal skills through social engagement. The therapeutic work in these areas fosters engagement in treatment through increased motivation and responsiveness by addressing contributing or underlying issues that have not been addressed by conventional treatment. **Figure 2** illustrates that these processes are not linear but rather circuitous, meaning an individual may move back and forth through these at different levels of ED treatment.

### Quantitative results

Overview of quantitative study characteristics (SN) included only five studies which met inclusion criteria (see **Table 2**, with one study included in **Tables 1**, **3** and **5** as a mixed methods study (71)). Three studies focused on patients with anorexia and two studies also included other EDs. Comorbid mental health diagnoses were reported in two studies. Subjects were 92-100% female (seven males out 169 total participants across studies), all but one study was conducted in adults, and all studies used a pre-post design. **Table 5** outlines the results of the quantitative studies, explicating study duration, design, music intervention and main outcomes. A Canadian study of group music therapy (MT, e.g. improvising and drumming) showed a significant decrease in negative affect and emotional suppression following MT; however, the MT also included mindfulness and was accompanied by treatment as usual (TAU) such as CBT and DBT (71). With this multifaceted intervention and no control group, one cannot discern whether the MT or other intervention components were instrumental in driving this observed improvement. Two Australian studies involved interventions comprised solely of group MT (e.g. singing and discussing songs), based on a humanistic therapeutic approach (48, 49). While neither study had random assignment, both interventions showed a significant decline in anxiety and distress immediately post intervention and one study showed a steeper decline in the intervention versus inpatient TAU control (48). A crossover study (where participants receive all conditions and so are their own control) of an MBI in Italian inpatients showed that compared with no music being played at supervised mealtimes, when patients listened to either classical or pop music during mealtimes, they consumed more food and had greater positive emotions following the meal (74). However, there was no waiting period between the interventions to ensure the effects of the first treatment wore off before delivering the next treatment (75).

**Table 5:** Results of the quantitative studies.

Author, Year	Study duration and design	MBI	Control condition	Other Interventions Received	Main Outcomes
Bibb et al., 2015 (48)	36-weeks, non-randomised pre-post design, pilot study	Group music therapy post mealtime, twice a week: singing, listening to songs, discussing music, and collaboratively writing songs	Inpatient treatment as usual: structured post meal support therapy which involved one-hour group sessions including games, art, discussions of feelings	None	Decrease in anxiety/distress in the intervention condition (pre-post M: 8 to 5.6) and control group (pre-post M: 8.1 to 7.1). Significant difference between the control group and intervention condition (p < 0.001).
Bibb et al., 2019 (49)	13-weeks, single group pre-post design	1 hour group music therapy (singing and song discussion) following a weekly group lunch at local cafe (social eating challenge) and before afternoon tea each week	No	None	Significant decrease in anxiety/distress following the intervention (M: 5.1 vs 2.9, p < 0.001).
Meneguzzo et al., 2024 (74)	4 weeks, within-subjects crossover design	Background music played during lunch and dinner in two formats: continuous classical piano music and preset pop music playlist. Each participant experienced each condition (including control) at least three times. Condition order was randomised	No-music during mealtime condition - only other interventions were received (see next column)	Psycho-nutritional rehabilitation. Supervised meals by dietitians and nurses Standardized meal composition and portioning. Monitoring of food intake, eating rituals, and adherence to meal timing. Encouragement of table conversation in all conditions	Both music conditions compared with no music showed increased food eaten (ps ⋦ 0.019) and post-meal positive emotions (p < 0.001). There was no significant difference between classical piano and pop music on these outcomes. Both music conditions were no different from the no-music condition on eaten carbohydrates, hunger, satiety, desire to eat, stress, and negative emotions
Shah et al., 2023 (71)	4-weeks, pre-post design, mixed methods qualitative results see **Table 3**, study characteristics see **Table 1**	Group music therapy sessions using a combination of improvisation, singing, drumming, listening back to recorded group improvisations and responding through visual art, and mindfulness-based exercises	No control group	Inpatient eating disorder treatment - largely group-based, including psychoeducation and psychotherapy sessions based on dialectical behaviour and cognitive behavioural therapy, creative arts programming, recreation and horticultural therapies	Participants experienced a decrease in negative affect (p = 0.006) and emotional suppression (p = .018) after a 4-week music therapy programme, compared to pre-intervention data. There was no significant overall change in positive affect or impulse control.
Wang and Xiao, 2021 (75)	3-months, RCT, pre-post design, per-protocol analysis excluded dropouts	Music therapy (MT) - patients were asked to listen to music related to health promotion in their daily life, chosen by the therapists based on the therapeutic method of combining Chinese and Western music	Conventional outpatient treatment including dietary adjustments and low-dose antipsychotic medications for insomnia or emotional issues	Conventional outpatient treatment as per control group, plus CBT for anorexia, adapted for Chinese setting (12 two-hour sessions)	Post treatment MT+CBT vs control: weight and BMI higher (p < 0.05), abdominal subcutaneous fat thicker (p < 0.05), eating disorder examination questionnaire scores lower (p < 0.05), Beck anxiety inventory and Beck depression inventory scores lower (p < 0.05), more participants satisfied with the intervention (p < 0.05).

Finally, a Chinese study on adolescents involved a receptive (music listening) MT intervention plus CBT and TAU compared with inpatient TAU only as the control group (76). While these groups were randomly assigned, the study conducted per-protocol analyses without dropouts, and there was a significantly greater dropout in the control vs intervention group (22.5% vs 5.4%). The study reported a greater increase in post-treatment weight, BMI, and abdominal subcutaneous fat in the MT + CBT arm compared with the control group. However, it is impossible to determine whether this was due to MT, CBT, or both. MBI reporting of the quantitative studies ranged from 55-82% with a mean of 67%, reflecting reporting on about 7 of the 11 components. While no negative outcomes were reported in any of the quantitative studies, null findings were reported in two of the studies (e.g. positive affect and impulse control (71); negative affect, stress, satiety (74)). In sum, coupled with small sample sizes, none of the quantitative studies implemented best practice of randomly assigning individuals to clear MBI and control groups, precluding firm conclusions about the efficacy of MBIs in people with EDs.

## Discussion

This scoping review examined a broad body of evidence allowing for a general synthesis of outcomes following MBIs for individuals in ED treatment. It included 20 studies that investigated MBIs for individuals engaged in ED treatment. This resulted in 16 studies that examined purely qualitative data (including case reports), four that assessed quantitative outcomes and one mixed methods study. Due to the specialized nature of ED treatment and the limited body of literature related to MBIs in ED treatment, this scoping review was conducted to capture and review a broader scope of research literature focused on MBIs as a primary intervention for ED, which differs from previous reviews (44–47). This helped to capture research with diverse methodologies as it included clinical case reports that are reflective of clinical practice and the therapeutic process. As a result, it helps contextualize and provide a more comprehensive understanding of the use of MBIs in research and in practice of music therapy in ED treatment (64, 78).

The nature of a scoping review is not to provide an overall assessment of quality of the literature, as this rigor can lead to missing or excluding relevant research (79). Capturing a diverse body of literature can provide deep and rich data to examine thorough descriptions of the therapeutic process (80). This scoping review examined studies published in English which precluded non-English language studies and as a result limits generalisability in some cultures (81, 82). Furthermore, our review only found one study which was not from a high-income Western country, and there were very few males studied, pointing to the importance of focused work in these groups. Given the majority of the literature reviewed included case studies, feasibility and pilot studies with small sample sizes and no control groups includes a risk of selection or information bias which limits the generalisability of the results (83). Further, it is worth noting that synthesizing the findings was challenging as therapeutic work within case reports is tailored and unique to each individual and at times a lack of clear and detailed information is provided when reporting the MBI. As a result, the findings are less directly relevant to clinical or treatment guidelines but can help to identify clinical considerations for treatment and inform recommendations for future research (50). Further, negative outcomes were only reported in one study and were specific to the use of the body monochord (BoMo) during inpatient ED treatment which resulted in experiencing the body more consciously and a negative evaluation of new experiences. Further, it is not clear what experience or training the therapist had with the BoMo as the therapist was not a qualified music therapist (59). These findings may indicate the need to identify at what point in the treatment trajectory is most appropriate for this type of body based therapeutic work. The limited reporting of negative outcomes across studies may indicate bias and a need for more transparent reporting. However, negative outcomes may be mitigated by music therapists’ reported awareness and training regarding avoiding harm in ED therapeutic settings (50, 84, 85).

### Music-based interventions utilised in eating disorder treatment

The studies implemented a wide array of MBIs, including music listening (listening to classical or popular music and song discussion, music-based relaxation, music-directed imaging, movement to music, and vibroacoustic stimulation), song writing, instrumental and vocal improvisation and active music making experiences (singing, group singing and drumming), while several studies integrated multiple types of MBIs. Although not reported in many of the included studies, theoretical approaches reported within the music therapy interventions included psychotherapeutic, cognitive-behavioural and trauma-informed music therapy. The large number of interventions utilised illustrates the diversity and depth of MBI options available to meet the emerging and changing needs of a patient at various levels of care and across the treatment process (63). These findings are consistent with a recent mixed methods study that surveyed and interviewed music therapists working with patients in ED treatment, which indicated clinicians utilize a wide array of methods in their regular clinical practice to address diverse therapeutic needs across levels of treatment and adapt these to meet the needs of individual patients and groups (50). This aligns with the focus on personalized ED care with tailored interventions based on individual psychological, biological, environmental, and genetic profiles (86).

While it is evident that the current body of research does not fully represent the diversity of interventions utilized by qualified music therapists and non-music therapists in the context of clinical practice in ED treatment, the inclusion of case reports (half of the studies in our review) gives breadth and depth of understanding and further highlights other methods including song discussion, music listening, group drumming, guided imagery and music, and music and movement. Additionally, it is worth noting that all but two of the studies in the scoping review (18/20) integrated music therapy facilitated by a qualified music therapist, highlighting a need for professional training required to implement MBIs within the treatment of these complex disorders.

Overall, the qualitative studies provided detailed information when reporting the MBI, meeting an average of 84% of MBI reporting guidelines (51, 53), compared to the quantitative studies that reported an average of 67% of the guidelines (note six qualitative studies were published before the MBI guidelines and all quantitative studies were published four or more years after publication of the MBI guidelines). Given that 10 of the 17 qualitative studies include case reports which typically provide rich and thorough descriptions of the therapeutic process, this difference in reporting is not surprising. Information consistently missing from the quantitative studies included details regarding the music intervention and materials utilized in the sessions. Clear and detailed reporting of MBIs is key to transparent reporting of the intervention and for replicating a study. Nonetheless, studies within this review demonstrate an improvement in the overall quality of reporting compared to a previous evaluation of music intervention studies, where reporting was poor (52). This may be due to the fact that more of the present studies were, which typically include detailed information about the music-intervention within the therapeutic process.

### Music-based interventions methods of delivery

In a majority of the studies in this review the MBIs were facilitated by a qualified music therapist. The other three studies included group singing workshops led by music teachers (68), vibroacoustic stimulation led by a therapist (no specific therapeutic training specified) (58) and music listening during mealtime delivered by dieticians and nurses (75). In the three studies facilitated by non-music therapists, participants reported numerous positive outcomes, with some negative outcomes related to the vibroacoustic body music. These studies illustrate how researchers are flexibly utilizing different ways of engaging with music to address the needs of patients in ED treatment. The reporting of the MBI in these three studies varied significantly, from 5 of the 11 aspects of the intervention (58) to 9 of the 11 (68). Ensuring that all MBI research adheres to the MBI quality reporting guidelines (51, 54) is instrumental, so the intervention is described in detail and there is clarity, and transparency in research methodology. The complex nature of music stimuli requires detailed and careful description to ensure a study can be replicated and necessitates music-based knowledge and expertise to describe it effectively. Since MBIs include music experiences that may be facilitated by music therapists, administered by staff, community musicians, other providers, or self-administered by the patient/patient, clarity in intervention descriptions is vital (50, 54). Dedication and advocacy to improve the quality of reporting of MBIs will help improve the rigor and reproducibility of these interventions (51, 52, 54, 85).

It is evident from our review that group and individual sessions offer unique affordances and may help to address different therapeutic needs. Across our reviewed studies (excluding case studies, which by necessity were individual MBIs), individual and group MBIs were used comparably (n=5 and n=4, respectively), with one study not reporting which method was used. Group based music interventions provide opportunities that capitalise on meeting new people, fostering a social connection (71), engaging in healthy social interactions, and feeling a sense of belonging (68), as well as feeling motivated to engage in treatment (64).

Group sessions can be economical and providing opportunities for social engagement in a shared or collaborative experience is particularly important if an individual feels isolated due to their ED (76). There is also a means of support that can be embedded or experienced in group MBIs that is valuable when engaging in a new or novel experience such as improvisation or songwriting (66, 73). Conversely, individual sessions can provide therapeutic space needed to address complex or deeply personal issues, which may include ED symptoms (restricting or purging) or trauma that are not appropriate for group based work (61, 86, 87). A trauma-informed approach to MBI practice is recommended to inform clinical decision-making and avoid causing harm (42, 50, 61). From synthesising information regarding patient presentation across the studies, we are encouraged that choice of group versus individual MBIs appear to have been selected to best meet patient needs.

### Addressing multiple and diverse issues in eating disorder treatment

EDs are complex in nature due to the multiple underlying factors that contribute to their development, the symptoms that negatively impact physical health and psychological wellbeing, a myriad of comorbid diagnoses, and a high incidence of trauma (15, 86, 87). While only half of the studies in our scoping review reported on comorbid mental health conditions, seven comorbid conditions (including trauma-related conditions) were reported across these studies. This indicates that many MBI studies include complex cases but also highlights that presence (or absence) of comorbid conditions needs more consistent reporting in future studies. This complex clinical matrix necessitates treatment across various levels of care that addresses the diverse and underlying issues needed to support an individual’s ED treatment process (36, 37, 62). The studies in this scoping review included individuals engaged in various levels of treatment including inpatient, residential, and outpatient. This is consistent with survey data indicating the clinical settings in which music therapists work with patients with EDs (50). While ED treatment may occur at specialty ED treatment programs, music therapists also provide services to patients in private practice, mental health units, medical units, long term psychiatric settings, and community mental health settings (46, 50). Providing treatment across various levels of care indicates that music therapy and MBIs are utilized to address a variety of therapeutic needs.

The present review’s qualitative and quantitative results indicate MBIs focus on a wide array of therapeutic issues and needs. The qualitative themes emerging from these interventions operate across various levels of ED treatment and recovery - that of symptom management, self-development, and treatment engagement as indicated by our model in **Figure 1**. The organization of these themes aligns with various levels of therapeutic work consistent with previous literature that highlights, “the flexibility of music as a therapeutic agent allows the therapist to individualize the process and meet a wide array of needs simultaneously, especially when feelings and emotions may be fragmented, elusive and inaccessible to language” (77) (p.128). Addressing core emotional processes is key, as impaired affective processes are often implicated in the maintenance of disordered eating behaviours (87). Recent research including a comprehensive synthesis of 30 years of clinical and empirical evidence underscores the importance of affective temperament, emotion regulation, and personality vulnerabilities related to symptom management (emotions, cognition, and body image) as a prerequisite to self-development (89–92). Thus, our model begins with symptom management by fostering emotional expression and learning coping skills to manage stress. This supports changes in cognition such as improved attention, and cognitive restructuring, which helps to decrease distortions. This supports shifting the relationship with one’s body by improved body awareness and image and increasing the level of comfort in one’s body through experiencing new ways of engaging the body.

In our model, symptom management then supports engaging in the next level of self-development which includes improved self-awareness, understanding, and self-esteem, which is substantiated by research indicating that addressing these emotional underpinning of the ED enhances treatment outcomes (88). Prior MBI literature highlights how songwriting links self-expression to the development of autonomy and exploring or developing one’s identity (66, 69). Building this self-capacity is key to a growth mindset which is necessary to support embracing challenges by facing fears and engaging in new experiences, which can foster hope and optimism (62, 63). These experiences are instrumental in having the capacity to develop interpersonal skills through increased social engagement, which helps to foster a sense of belonging and engagement in meaningful relationships (61, 65). This work can then lead to increased motivation to engage in treatment, fostering responsiveness to address and target contributing issues that have not been addressed in conventional ED treatment (44, 46, 62). However, we note that our model follows an iterative feedback loop, with treatment engagement facilitating further symptom management and self-development, which promotes continued healing and recovery. We further acknowledge that effective initial symptom management must be catalysed by an initial willingness to engage in treatment.

The capacity of MBIs to address diverse therapeutic needs across various levels of treatment is also highlighted in Dvorak’s survey of music therapists working in ED treatment (50). These therapists reported addressing 90 different treatment areas, with the five most common including self-expression, development of coping skills, managing mood and depression, and identifying and expressing feelings. While these only encompass the first component of the “symptom management” level in our model, cognition and body image/awareness (other components of the “symptom management” level) were treatment areas identified by more than half of therapists in that study. Further, self-esteem and self-awareness (operating on the “self-development” level of our model) were identified by therapists as comparably frequent treatment areas to identifying and expressing feelings (50). However, interpersonal/social aspects and optimism (other components of the “self-development” level) were less frequently cited as treatment areas by therapists in the study, and growth mindset components of embracing challenge and novelty-seeking were not included as a response in this survey. This highlights the importance of qualitative research which can bring out important factors which have been previously unidentified. Our review reveals growth mindset as a potentially important outcome which should be assessed in future MBIs for people with EDs. Indeed, previous research has explicated the unique capacity of creative and aesthetic experiences to afford opportunities to externalize and explore their thoughts and feelings in new and different ways, which helps an individual to develop new understandings and attach new meaning to them (63, 93, 94). This is further evidenced by quotes from **Table 4** which indicate that making music (nonverbal) serves as a referent to project, explore or recast one’s inner experiences (65) and that this process of externalizing inner experiences helps to make meaning and comprehend their experiences (62). Engagement in a supported creative aesthetic therapeutic experience can help individuals in ED treatment access affective, cognitive and perceptual brain functions which are key to their treatment and recovery process (92) and are instrumental in developing insight, fostering growth, and a sense of mastery and self-efficacy (62, 63, 66, 72, 73).

### Implications of this study and recommendations for future research

Although findings generally support positive outcomes for individuals ED treatment following a MBI, our conclusions are impeded by the heterogeneity of interventions and outcomes, and inadequately controlled methods to ascertain effectiveness. Synthesizing this body of literature is also impacted by the complex nature of EDs, including the physical and psychological aspects of the disorder, as well multiple comorbid diagnoses and trauma history. While some research methodologies may not be able to robustly capture the nuanced and complex phenomenon of MBIs, ensuring clear and transparent reporting is necessary to provide a clear understanding of the therapeutic intervention. Further, these shortcomings will be addressed with more rigorous quantitative studies which capture the range of patients treated in practice and appropriately assess treatment effects. To that end, there is a particular need for well-powered randomised controlled trials with distinct and clearly defined comparison groups, implementing intention-to-treat analyses and assessment of and controlling for diverse clinical and background factors (96). This will necessitate researchers addressing the challenges and barriers in conducting RCTs with MBIs in ED treatment, particularly balancing the personalisation of therapy with the required standardisation (97).

Future reviews should aim to understand and explicate the efficacy, benefits, and risks of MBIs in ED treatment, and the role of MBIs in addressing the complex and myriad needs of individuals in the treatment process. Given the established link between emotional rigidity, emotion regulation, and ED symptoms, there is need for specific exploration of MBIs that support the development of temperament-linked coping styles (50, 78, 89). Further, all future studies should provide detailed information on case presentation (including comorbidities), comprehensive descriptions of the MBIs utilized, as well as the experience, qualifications, and theoretical orientation of the professionals facilitating the MBI. Future reviews should assess each study’s adherence to MBI quality reporting guidelines (51, 54). Due to the unique nature, variety, and complexity of MBIs, adhering to these reporting guidelines to explicate the different components of the intervention is crucial in supporting replicability and identifying key and active elements in the intervention (44, 51, 52, 54).

## Conclusion

The high economic cost and incidence of EDs indicates a need for innovative and effective treatment approaches (97, 98). While there is a small corpus of literature to date on MBIs for EDs, there is clear evidence that MBIs have the capacity to address a wide array of therapeutic needs for individuals across the various levels of ED treatment. The present review shows across a range of case reports and research studies that MBIs can be tailored to individual or group therapy to meet emerging and changing needs over the course of ED treatment. The unique potential of music therapy to not only address a myriad of issues but also uncover issues that have not been addressed in verbal therapy suggests that ED treatment programs would benefit from integrating this type of creative modality alongside psychological therapies. Further rigorous quantitative studies, especially randomised controlled trials evaluating and comparing MBI enhanced therapy to more traditional approaches are required to evidence support of more widespread usage of MBI for people living with ED. This type of evidence is necessary to inform clinical treatment guidelines (i.e. NICE guidelines), help to promote government support for funding, and foster widespread usage of these modalities (95).

## Data Availability

The original contributions presented in the study are included in the article/supplementary material. Further inquiries can be directed to the corresponding author.
